# Impact of electronic cigarette use and sleep duration, sleep issues and insomnia: a systematic review and meta-analysis

**DOI:** 10.3389/fpubh.2025.1662234

**Published:** 2025-08-29

**Authors:** Huma Sulthana, Asif Jan, Amogh Verma, Ranjana Sah, Rachana Mehta, Aftab Ullah, Abdur Rahim, Mohammad R. Alqudimat, Asmat Ullah

**Affiliations:** ^1^Department of Pharmaceutical Chemistry, Faculty of Pharmacy, M. S. Ramaiah University of Applied Sciences, Bangalore, India; ^2^Saidu Group of Teaching Hospitals, Saidu Sharif, Pakistan; ^3^Department of Pharmacy, University of Peshawar, Peshawar, Pakistan; ^4^District Headquarter Hospital-Charsadda, Charsadda, Pakistan; ^5^Department of Internal Medicine, Rama Medical College Hospital and Research Centre, Hapur, India; ^6^Department of Paediatrics, Dr. D. Y. Patil Medical College Hospital and Research Centre, Dr. D. Y. Patil Vidyapeeth (Deemed-to-be-University), Pune, India; ^7^Department of Public Health Dentistry, Dr. D. Y. Patil Dental College and Hospital, Dr. D. Y. Patil Vidyapeeth (Deemed-to-be-University), Pune, India; ^8^Clinical Microbiology, RDC, Manav Rachna International Institute of Research and Studies, Faridabad, India; ^9^Department of Pharmacy, Abasyn University Peshawar, Peshawar, Pakistan; ^10^Department of Pharmacy, CECOS University Peshawar, Peshawar, Pakistan; ^11^American University of the Middle East, Kuwait City, Kuwait; ^12^Spinghar University Faculty of Medicine, Jalalabad, Afghanistan

**Keywords:** electronic cigarettes, insomnia, sleep duration, sleep issues, health problems

## Abstract

**Background:**

The increasing popularity of electronic cigarettes (e-cigarettes) has introduced new public health challenges and concerns. While promoted as safer alternatives to conventional tobacco and as tools for quitting smoking, e-cigarettes have raised alarm about possible long-term health consequences. This systematic review and meta-analysis sought to evaluate the association between electronic cigarette consumption and sleep disturbances.

**Methods:**

We performed comprehensive searches in EMBASE, Web of Science, and PubMed up to September 18, 2024, to locate studies examining the link between e-cigarette use and sleep duration, sleep disorders, and insomnia. A meta-analysis was conducted to calculate pooled odds ratios (ORs). The quality of the studies was evaluated using the Newcastle-Ottawa Scale. Meta-analysis was performed using R software (Version 4.3).

**Results:**

A total of 14 cross-sectional studies were included from 554 unique records screened. E-cigarette users exhibited a notably elevated risk of having shorter sleep duration compared to non-users, with a pooled odds ratio of 1.38 (95% CI: 1.24–1.55). Several studies reported that e-cigarette users also had higher odds of sleep disturbances, such as insomnia and reliance on sleep medications. Among adolescents, e-cigarette use was associated with a 33 to 61% increased risk of inadequate sleep.

**Conclusion:**

E-cigarette use may be associated with sleep disturbances, including shorter sleep duration and increased sleep difficulties. Future long term longitudinal studies are warranted for better evidence.

## 1 Introduction

Electronic cigarettes (e-cigarettes) are devices that vaporize a liquid containing nicotine, flavorings, and other chemicals, simulating a smoking-like experience without combustion (1). Initially marketed as a safer alternative to traditional tobacco products, e-cigarettes have gained significant popularity (2, 3). However, increasing evidence suggests that their use may be associated with various health issues. Research has highlighted potential risks associated with exposure to e-cigarette aerosols, including impacts on pulmonary function, cardiovascular well-being, and sleep quality (4–7).

Research indicates that nicotine, a primary component in most e-cigarettes, disrupts sleep patterns due to its stimulating properties, leading to difficulty falling asleep and maintaining restful sleep (8, 9). In addition, inhaling e-cigarettes may affect lung function and cause oxidative stress, further contributing to impaired sleep quality (10, 11). Research indicates that e-cigarette use is associated with negative sleep outcomes, including inadequate sleep duration and trouble sleeping. The use of e-cigarettes has been linked to negative effects on sleep health, including increased odds of inadequate sleep duration and trouble sleeping. Research indicates that both current and former e-cigarette users experience significant sleep disturbances compared to non-users. Current e-cigarette users are 1.82 times more likely to experience shorter sleep durations than non-users (12). Among young adults, exclusive e-cigarette users have an increased odds of inadequate sleep duration after adjusting for various co-variates (13). Dual users of e-cigarettes and conventional tobacco report increased sleep latency, taking longer to fall asleep (14). In addition to that, e-cigarette users have reported greater sleep difficulties and a higher reliance on sleep medications compared to non-users (15).

The association between e-cigarette use and poor sleep health is explored by some studies with varying results (16). However, some research suggests that individual factors may influence this relationship, highlighting the complexity of interactions between e-cigarette use and sleep health (15, 16). Despite their growing use, the long-term impact of e-cigarettes on sleep remains understudied, and further research is needed to understand the extent of these disturbances.

A systematic review on this topic has not yet been conducted. Therefore, this systematic review and meta-analysis aim to bridge this research gap by examining the existing literature on the link between e-cigarette use and sleep disturbances. Through the synthesis of findings from various studies, the analysis seeks to offer a detailed understanding of this association. The findings are anticipated to offer valuable insights into the potential psychosocial risks associated with e-cigarette use, laying the groundwork for further studies and informing policymakers and healthcare providers in mitigating the harmful effects of e-cigarette usage.

## 2 Methods

This systematic review and meta-analysis complied with the PRISMA (Preferred Reporting Items for Systematic Reviews and Meta-Analyses) guidelines to guarantee both clarity and comprehensiveness in its reporting methodology (Supplementary Table S1). The primary goal was to systematically evaluate and consolidate the current evidence on the correlation among e-cigarette usage, sleep duration, sleep disturbances, and insomnia. Additionally, this review has been pre-registered in the PROSPERO.

### 2.1 Search strategy

A structured search strategy was formulated to locate studies examining the link among e-cigarette consumption and sleep duration, sleep disturbances, and insomnia. A thorough search of various electronic databases, including EMBASE, Web of Science, and PubMed, was carried out from their inception up to September 18, 2024. The search method employed a combination of terms related to e-cigarette use (e.g., “vaping,” “electronic nicotine delivery systems,” “e-cigarettes”) along with terms associated with sleep disorders (e.g., “insomnia,” “sleep disturbances,” “sleep–wake disorders,” “sleep apnea,” and “sleep disorder”). A detailed description of the search strategy is provided in Supplementary Table S2.

### 2.2 Eligibility criteria

Studies involving the general population were included without restrictions to allow for a wide-ranging and thorough analysis. The main exposure of interest was identified as the use of e-cigarettes, vaping devices, or Electronic Nicotine Delivery Systems (ENDS). However, studies that solely concentrated on conventional tobacco smoking were excluded due to its distinct mechanisms and well-documented effects. In terms of outcomes, we incorporated studies investigating sleep disorders, sleep patterns, abnormal dreams, sleep disturbances, sleep–wake disorders, sleep apnea, parasomnia, difficulties with sleep initiation and maintenance, dyssomnia or insomnia. Eligible study designs included observational studies (cross-sectional, longitudinal, retrospective, prospective, and case–control) to capture the available quantitative evidence on e-cigarette use and sleep disturbances. No experimental studies, such as randomized controlled trials, were identified in our comprehensive search, likely due to the ethical constraints of exposing participants to e-cigarettes and the emerging nature of this research field. To minimize confounding from pre-existing conditions that may independently affect sleep quality, we excluded studies focusing on populations with specific chronic diseases. Qualitative research, policy evaluations, commentaries, case reports, case series, systematic reviews, and animal studies were excluded, as these sources lacked the empirical data necessary to directly answer our research question.

### 2.3 Study selection

Two reviewers independently screened the titles and abstracts for eligibility using the Nested-Knowledge web platform. Full-text articles of studies identified as potentially relevant were procured and independently scrutinized for eligibility. Any discrepancies were resolved through deliberation or, when required, by consulting a third reviewer.

### 2.4 Data extraction

Data extraction was performed independently by two reviewers utilizing a standardized template. The extracted data encompassed study attributes (e.g., author, publication year, country, study design, and sample size), participant demographics, and sleep disorder outcomes. Any discrepancies identified during the extraction process were resolved through deliberation or, if necessary, by consulting a third reviewer.

### 2.5 Quality assessment

The quality evaluation of the studies was performed through the Newcastle-Ottawa Scale, which reviews the cohort selection process, the comparability of the cohorts, and how the exposure or outcome of interest was assessed.

### 2.6 Statistical analysis

A random effects model was selected for the meta-analysis to account for the heterogeneity in study populations, e-cigarette use patterns, and sleep outcome measures, which likely contribute to variability in effect sizes across studies. This approach allows for a more conservative estimate of the pooled effect while acknowledging between-study differences. The link between e-cigarette use and sleep disorders was analyzed using odds ratios (ORs) with 95% confidence intervals (CIs) (17, 18). The *I*^2^ statistic was employed to quantify heterogeneity across the studies, with a 95% prediction interval providing additional insight into the extent of variability. Publication bias was evaluated through funnel plots and Egger’s test. The analysis was conducted using the ‘Meta’ and ‘Metafor’ packages in R statistical software (Version 4.3).

## 3 Result

### 3.1 Literature search

**Figure 1:**
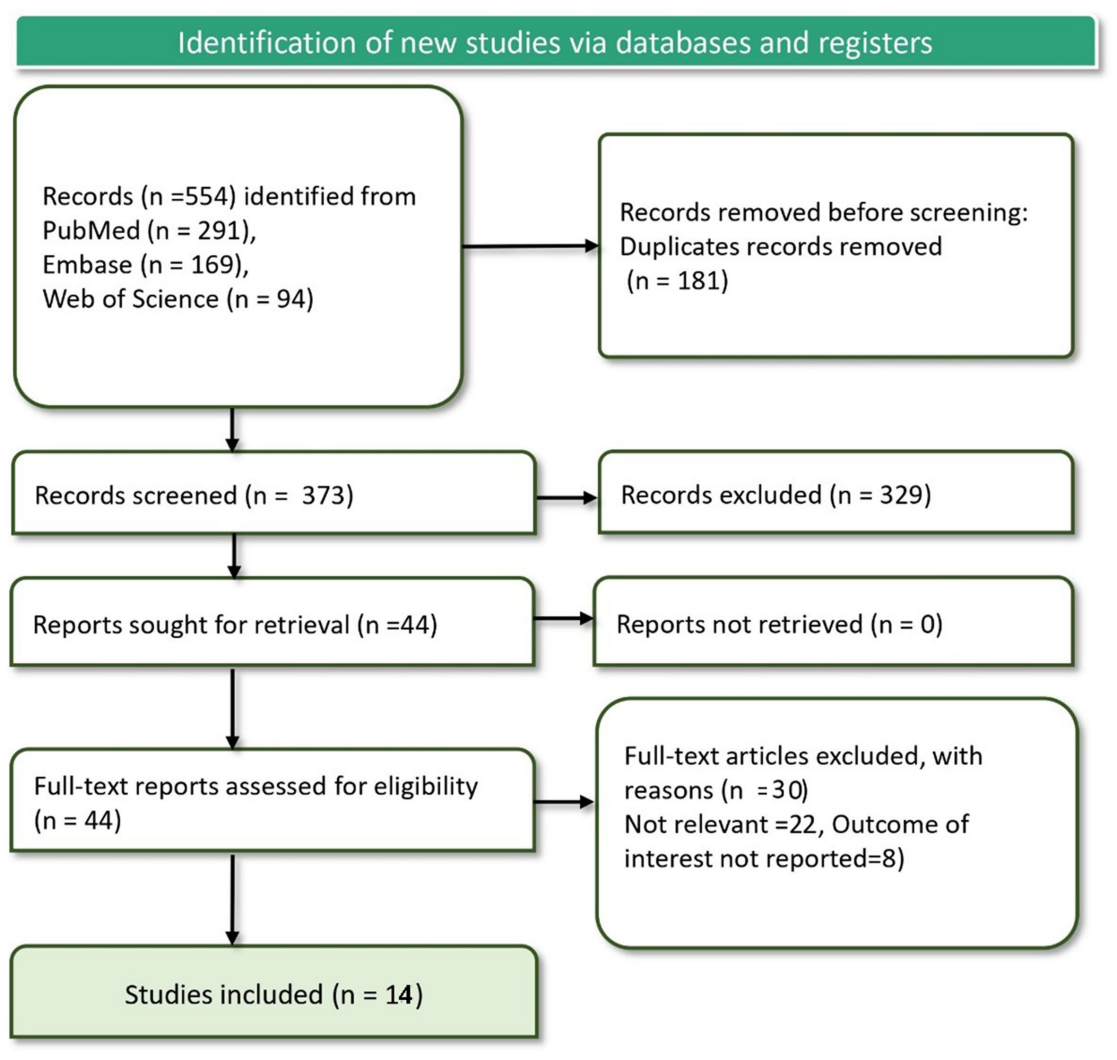
PRISMA flow diagram showing article screening and study selection process.

The primary search identified a total of 554 records, which was narrowed down to 373 after eliminating 181 duplicates. Each remaining record was evaluated according to our established inclusion criteria, resulting in 44 reports being deemed potentially relevant for full-text review. However, a detailed assessment led to the exclusion of 30 reports, primarily because they did not address the desired outcomes, specifically sleep disturbances in relation to e-cigarette consumption. Ultimately, 14 studies were found to be eligible (12, 15, 16, 19–29) for inclusion in our systematic review and meta-analysis (Figure 1).

### 3.2 Characteristics of included studies

This systematic review includes 14 cross-sectional studies across (Table 1) various countries, primarily in the USA, except for a few from Palestine, Korea, and Thailand. The sample sizes of these studies range from 227 to 179,004 participants. These studies utilized a cross-sectional design to examine the connection among e-cigarette use and sleep-related outcomes among various demographic groups, such as teenagers, college students, adults, and the general population. The studies consistently show that e-cigarette users experience poor sleep outcomes, such as reduced sleep quality, shorter sleep duration, and sleep disturbances. Adolescents and young adults are frequently impacted, with vaping linked to sleep deprivation, insomnia, and increased reliance on sleep medications. Multiple studies highlighted the association between e-cigarette use and insufficient sleep duration. The evaluation of study quality is presented in Supplementary Table S3.

**Table 1:** Characteristics of included studies.

Author	Study design	Country	Male %	Sample size	Type of Population	Outcome	OR (95% CI) for sleep-related problems	Key findings
Baiden 2023 (19)	Cross-sectional	USA	48.8	28,135	Adolescent Participants of Youth Risk Behavior Survey	Insufficient sleep	1.33 (1.16–1.52)	The use of EVPs was significantly associated with inadequate sleep duration among adolescents, even after controlling for demographic factors and other covariates.
Brett 2020 (15)	Cross-sectional	USA	33.7	1,664	College students	Sleep quality, sleep latency, sleep duration, sleep disturbances	NA	E-cigarette users exhibited a significantly higher prevalence of sleep disturbances, increased reliance on sleep medications, and poorer overall sleep quality.
Kianersi 2021 (20)	Cross-sectional	USA	54	19,701	Behavioral Risk Factor Surveillance System respondents aged between 18 and 24	Sleep deprivation	NA	E-cigarette users were more likely to be sleep deprived [PR: 1.42 (1.23–1.65)] than the non-users
Lee 2021 (48)	Cross-sectional	Korea	NA	52,928	Adolescents who responded to sleep satisfaction and sleep duration survey	Insufficient Sleep duration and sleep satisfaction	Less satisfaction with sleep = 1.36 (1.03–1.80), insufficient Sleep duration = 2.06 (1.40–3.05)	E-cigarette users were more likely to be dissatisfied with sleep and had lesser sleep duration in comparison to non-users
Mahamid 2022 (22)	Cross-sectional	Palestine	23	506	Participants of an online survey	Insomnia	NA	Insomnia was positively correlated with electronic cigarette smoking
Merianos 2021 (23)	Cross-sectional	USA	46.4	11,296	High school student	Insufficient sleep (<7 h/night)	1.61 (1.16–2.24)	E-cigarette users reported insufficient sleep more than non-users.
Riehm 2019 (24)	Cross-sectional	USA	51.3	9,588	Adolescents from the Population Assessment of Tobacco and Health Study	Sleep-related complaints (bad dreams, sleeping restlessly, or falling asleep during the day)	1.29 (1.05–1.59)	E-cigarette users were significantly associated with sleep-related complains than non-users.
So 2021 (25)	Cross-sectional	USA	45.8	227	E-cig users	Sleep onset latency, sleep duration, need medication to sleep, PSQI score	NA	E-cigarette frequency uniquely predicted increased daytime dysfunction due to sleepiness.
Thepthien 2023 (26)	Cross-sectional	Bangkok	51.2	5,740	High School Students	Sleep duration (5–6 h)	1.5 (1.3–1.8)	Lack of adequate sleep was noted more commonly in E- cigarette users in comparison to non-users.
Wang 2024 (16)	Cross-sectional	USA	48.6	11,659	National Health and Nutrition Examination Survey participants (adults)	Short sleep duration, trouble sleeping	Short sleep duration = 1.33 (0.85, 2.08), trouble sleeping = 2.16 (1.49–3.13)	E-cigarette use was associated with higher odds of trouble sleeping in comparison to non-users
Wiener 2020 (12)	Cross-sectional	USA	49.3	2,889	National Health and Nutrition Examination Survey participants	Less sleep duration	1.82 (1.18, 2.79)	E-cigarette smokers were significantly associated with lesser sleep duration in comparison to non-smokers.
Wilson 2024 (27)	Cross-sectional	USA	36.7	1775	College students	Restful nights of sleep/week	NA	Individuals who used e-cigarettes reported experiencing a reduced number of nights with adequate restful sleep and exhibited higher levels of alcohol consumption.
You 2023 (28)	Cross-sectional	Korea	48.6	179,004	Korean Community Health Survey participants	Sleep duration (<7 h/day)	1.31 (1.11–1.54)	E- cigarette users reported decreased sleep duration and depressive symptoms more in comparison to non-smokers
Zhu 2023 (29)	Cross-sectional	USA	48.6	11,248	National Health and Nutrition Examination Survey participants	Obstructive sleep apnea	1.14 (0.65–1.99)	No significant association between e-cigarette use and Obstructive sleep apnea

CI Confidence Interval, NA Not Available, OR Odds Ratio, EVPs electronic vapor products.

The association between e-cigarette use and sleep issues has been widely examined in these included studies revealing a consistent link between e-cigarette use and various sleep disturbances. Several studies, including Baiden et al. (19) and Merianos et al. (23), found that adolescent e-cigarette users were significantly more likely to experience insufficient sleep duration, with OR of 1.33 (95% CI: 1.16–1.52) and 1.61 (95% CI: 1.16–2.24), respectively. Similarly, Brett et al. (15) and Wilson (27) noted that college students who used e-cigarettes reported poorer sleep quality, greater reliance on sleep medications, fewer restful nights, and higher rates of sleep disturbances. Wang et al. (16) found an increased risk of trouble sleeping among e-cigarette users (OR: 2.16, 95% CI: 1.49–3.13). Similar findings were observed by Wiener (12), who linked e-cigarette use to shorter sleep durations (OR: 1.82, 95% CI: 1.18–2.79). Studies also showed that e-cigarette users experienced broader sleep-related complaints. For example, Riehm et al. (24) found that adolescents using e-cigarettes reported more frequent sleep-related issues such as bad dreams and daytime sleepiness (OR: 1.29, 95% CI: 1.05–1.59), while So (25) noted that e-cigarette use predicted increased daytime dysfunction due to sleepiness. In You et al. (28) reported similar associations in Korean adolescents, with e-cigarette users experiencing lower sleep satisfaction and shorter sleep durations (OR: 2.06, 95% CI: 1.40–3.05) and a higher likelihood of insufficient sleep (OR: 1.31, 95% CI: 1.11–1.54). Thepthien (26) observed similar results among high school students in Bangkok (OR: 1.5, 95% CI: 1.3–1.8).

### 3.3 Meta-analysis of the association between e-cigarette use and sleep duration

**Figure 2:**
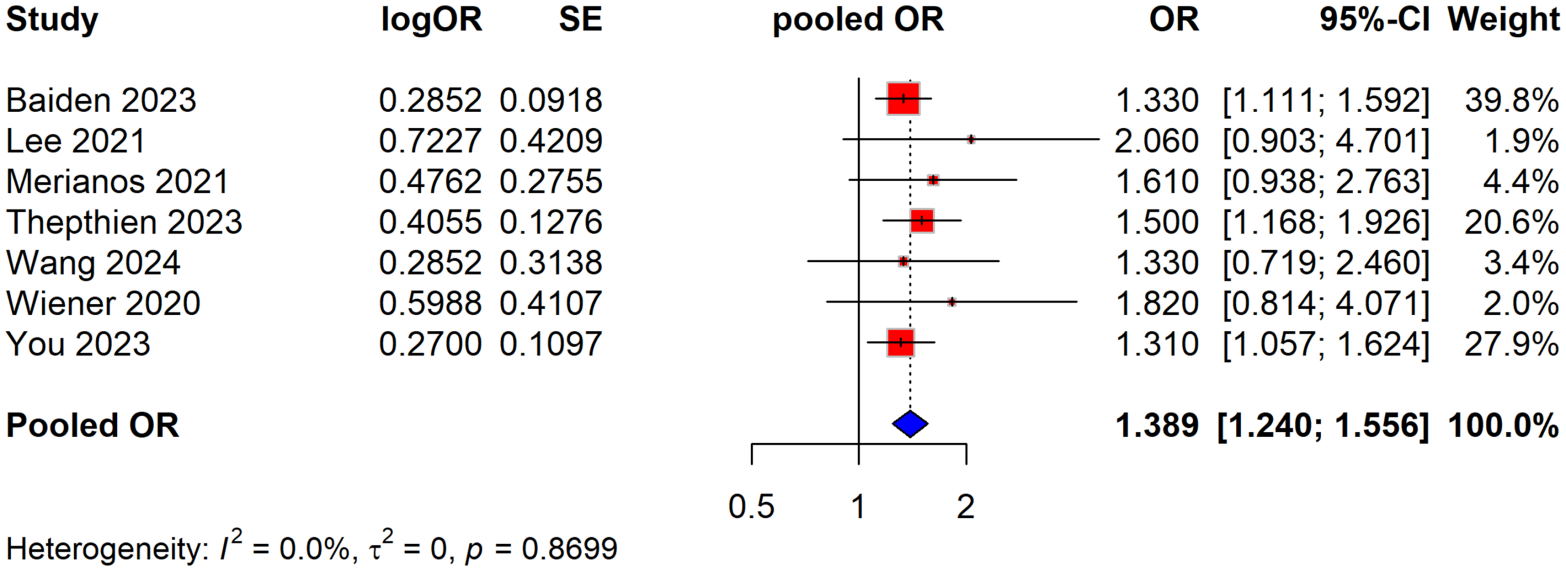
Association between electronic cigarette use and short sleep duration shown by pooled ORs from studies.

We performed a meta-analysis to evaluate the association between e-cigarette usage and inadequate sleep. The pooled odds ratio (OR = 1.38, 95% CI: 1.25 to 1.52) indicates a statistically significant association between insufficient or short sleep and e-cigarette smoking, suggesting a 38% increased risk in those who smoke e-cigarettes. The lack of heterogeneity (*I*^2^ = 0%) indicates that the results are consistent across the included studies. Thus, insufficient sleep appears to be a significant risk factor for the outcome of interest in this meta-analysis (Figure 2).

### 3.4 E-cigarette use and obstructive sleep apnea

Only one study reported the association between e-cigarette use and obstructive sleep apnea (29). The study found no significant association, with an OR of 1.14 (95% CI: 0.65–1.99).

### 3.5 Sensitivity analysis

**Figure 3:**
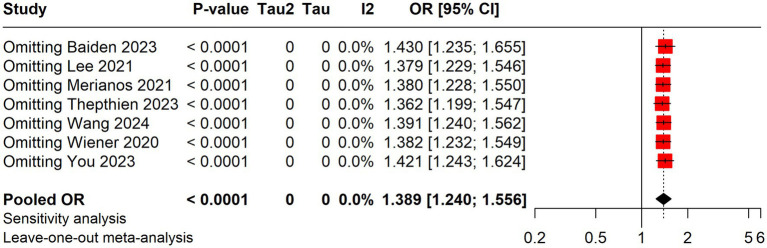
Sensitivity analysis.

A sensitivity analysis was performed utilizing the leave-one-out method, showing that the overall results of the meta-analysis are robust. No individual study substantially impacts the pooled OR, as the OR values remain consistent across the different leave-one-out scenarios. Thus, the overall odds ratio can be considered reliable and consistent even when individual studies are excluded from the analysis (Figure 3).

### 3.6 Publication bias

**Figure 4:**
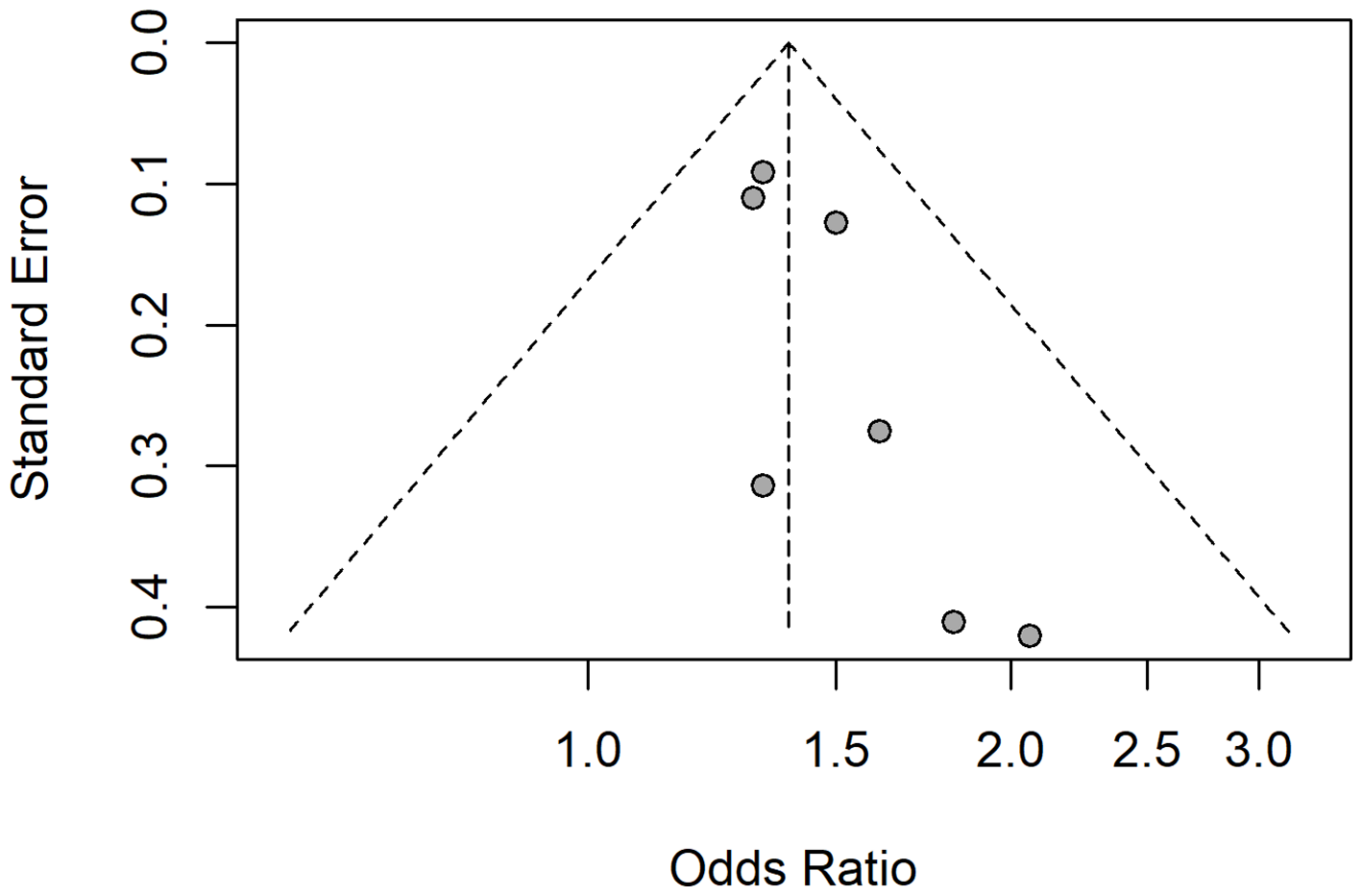
Funnel plot assessing presence of publication bias.

Publication bias in this meta-analysis was assessed through graphical examination of funnel plots and the application of Egger’s test, as presented in Figure 4. In the funnel plot, there appears to be a slight asymmetry, with more studies clustered on the right side, showing higher odds ratios. The left side contains fewer studies, which may suggest the potential for publication bias. However, the overall spread of the studies does not indicate extreme bias. The Egger test also indicated the possibility of publication bias, with a *p*-value of 0.03.

## 4 Discussion

The results of this study offer substantial documentation of a strong link between e-cigarette use and an increased risk of sleep disturbances, including shorter sleep duration, extended sleep latency, and reduced sleep quality. This is particularly concerning given the growing popularity of e-cigarettes, especially among adolescents and young adults. Our analysis shows that e-cigarette users are 39.1% more likely to suffer from inadequate sleep duration. These findings were consistently observed across studies involving both the general population and younger groups, suggesting that the connection between e-cigarette use and sleep problems is widespread and not limited to specific subpopulations.

Sleep is critical for both the physical and mental health of individuals, influencing nearly all aspects of a person’s life either directly or indirectly. Sleep has been given equal importance as healthy diet and physical workout for the better health of individuals (30). Researchers have emphasized the need of promoting importance of sleep health at community level globally (31). Inadequate sleep can affect the immune system thus making sleep deprived individuals more prone to various disease and infections (32, 33). Inadequate sleep for a longer duration can affect mental health of an individual leading to more depression and other related problems (34) Sleep is vital to maintain optimal health and proper day to day functions in our life. As proper sleep is crucial to maintain good health and functioning of body, any sleep related disturbance can not only effect normal physiological functioning of body but also affect various other aspects of individual’s life. Deficiency of sleep can affect productivity and economic growth of individual as well as the nation (35). Lack of adequate sleep can also lead fatigue and thus more road traffic accidents (36). The amount of sleep required for optimal health varies between individuals and is influenced by factors such as age and sociodemographic characteristics. Generally, 7 or more hours of sleep per night is considered necessary for maintaining good health (37). Insufficient sleep can lead to depression and physical activities/health is also affected (28, 38).

E-cigarette use, commonly referred to as vaping, has gained popularity over the past decade, particularly among adolescents and young adults. Recent research has begun to explore the potential health impacts of vaping, including its association with sleep disturbances. Electronic cigarettes, initially promoted as an alternative to conventional cigarettes, have been repeatedly shown to pose significant risks to physical, physiological and mental health (6, 26, 39). Nicotine, the primary addictive component of most e-cigarettes, is a stimulant that affects sleep quality. Nicotine activates β2-containing nAChRs, which are crucial for regulating sleep–wake cycles and micro-arousals. This activation can lead to increased wakefulness and disrupted sleep patterns (40, 41). In studies, nicotine exposure has been shown to diminish protective arousal responses during sleep, exacerbating conditions like sleep-disordered breathing (42). Smokers exhibit higher nocturnal hypoxia indices, indicating that e-cigarette use may similarly impair oxygen saturation during sleep, potentially leading to sleep apnea (43). The correlation between smoking intensity and decreased nocturnal oxygen saturation suggests that e-cigarette users may experience similar respiratory disturbances (44). While causality has yet to be fully established, the data suggest that the stimulant effects of nicotine combined with the other potential irritants in e-cigarette vapor may play a role in negatively affecting sleep. A dose–response relationship exists, where increased smoking correlates with heightened sleep disturbances, including snoring and short sleep (45, 46).

Our study demonstrates that e-cigarette use is associated with various sleep-related disturbances, including reduced sleep duration, increased sleep latency, poor sleep quality, and insomnia. The findings reveal a notable association between e-cigarette use and reduced or insufficient sleep duration, comparable to the harmful effects associated with traditional cigarette smoking (15). Even irregular e-cigarette users experience negative impacts on sleep, including shortened sleep duration and altered sleep quality. Sleep quality encompasses four key attributes: sleep efficiency, sleep latency, sleep duration, and wakefulness after sleep onset. Multiple studies have consistently reported decreased sleep duration and overall poorer sleep quality among e-cigarette users sleep (15, 47). Furthermore, e-cigarette users are more likely to use medications to aid sleep, and conditions such as insomnia and parasomnias are more prevalent in this group compared to non-users (22, 24, 48). Given the critical role of sleep in maintaining overall health, these findings emphasize the need for targeted interventions to reduce e-cigarette use and raise awareness about its impact on sleep health.

A key strength of our study is that significant associations were observed between e-cigarette use and sleep disturbances for most outcomes, with the exception of OSA, where findings were inconclusive. The consistency of the included studies is homogenous. Most of the included studies are of good quality. Our study has some limitations. A key limitation of this review is the reliance on cross-sectional studies, which limits the ability to establish temporality or causality between e-cigarette use and sleep disturbances. The cross-sectional nature of the included studies limits the ability to fully account for these confounders. While some studies adjusted for covariates like age, sex, and smoking status, residual confounding from unmeasured factors, such as psychological conditions or socioeconomic disparities, may persist. The absence of experimental studies in the literature highlights a critical gap, and future research should consider longitudinal or, where ethically feasible, experimental designs to better elucidate the causal pathways. Most of the studies are based in the United States, which may restrict the applicability of the results to populations in other regions. The potential for publication bias also raises concerns regarding the validity of the results. Our analysis might have overlooked important demographic factors that might impact the link between e-cigarette use and sleep disturbances, factors like economic background or underlying psychological conditions.

## 5 Conclusion

E-cigarettes are often considered as an alternative to conventional cigarettes; however, they are not devoid of harmful effects. Our study clearly highlighted the association between use of e-cigarettes and sleep related disturbances. Adequate sleep is fundamental to overall health and influences various aspects of life, both professional and personal. The use of e-cigarettes should be regulated, with increased public health initiatives and policy interventions aimed at reducing their consumption. Future longitudinal and, where feasible, experimental studies are warranted to establish temporality and causality, addressing the current reliance on observational data accounting for potential confounders, sociodemographic and lifestyle factors.

## Data Availability

The raw data supporting the conclusions of this article will be made available by the authors, without undue reservation.
