# Supplementary material for: Impact of electronic cigarette use and sleep duration, sleep issues and insomnia: a systematic review and meta-analysis
Source: Front Public Health. 2025 Aug 29;13:1662234. doi: 10.3389/fpubh.2025.1662234 (PMC12426188; doi:10.3389/fpubh.2025.1662234)
Supplement: Supplementary file 1 [file Table_1.DOCX]

**SUPPLEMENTARY MATERIALS**

**Table S1.** PRISMA Checklist

| **Section and Topic** | **Item #** | **Checklist item** | **Location where item is reported** |
| --- | --- | --- | --- |
| **TITLE** | | |  |
| Title | 1 | Identify the report as a systematic review. | 1 |
| **ABSTRACT** | | |  |
| Abstract | 2 | See the PRISMA 2020 for Abstracts checklist. (madeas per the Journal guidelines) | 2 |
| **INTRODUCTION** | | |  |
| Rationale | 3 | Describe the rationale for the review in the context of existing knowledge. | 3 |
| Objectives | 4 | Provide an explicit statement of the objective(s) or question(s) the review addresses. | 3 |
| **METHODS** | | |  |
| Eligibility criteria | 5 | Specify the inclusion and exclusion criteria for the review and how studies were grouped for the syntheses. | 3 |
| Information sources | 6 | Specify all databases, registers, websites, organisations, reference lists and other sources searched or consulted to identify studies. Specify the date when each source was last searched or consulted. | 3 |
| Search strategy | 7 | Present the full search strategies for all databases, registers and websites, including any filters and limits used. | Table S2 |
| Selection process | 8 | Specify the methods used to decide whether a study met the inclusion criteria of the review, including how many reviewers screened each record and each report retrieved, whether they worked independently, and if applicable, details of automation tools used in the process. | 4 |
| Data collection process | 9 | Specify the methods used to collect data from reports, including how many reviewers collected data from each report, whether they worked independently, any processes for obtaining or confirming data from study investigators, and if applicable, details of automation tools used in the process. | 4 |
| Data items | 10a | List and define all outcomes for which data were sought. Specify whether all results that were compatible with each outcome domain in each study were sought (e.g., for all measures, time points, analyses), and if not, the methods used to decide which results to collect. | 4,Table 1 |
|  | 10b | List and define all other variables for which data were sought (e.g., participant and intervention characteristics, funding sources). Describe any assumptions made about any missing or unclear information. | 4 |
| Study risk of bias assessment | 11 | Specify the methods used to assess risk of bias in the included studies, including details of the tool(s) used, how many reviewers assessed each study and whether they worked independently, and if applicable, details of automation tools used in the process. | 4, |
| Effect measures | 12 | Specify for each outcome the effect measure(s) (e.g. risk ratio, mean difference) used in the synthesis or presentation of results. | 5 |
| Synthesis methods | 13a | Describe the processes used to decide which studies were eligible for each synthesis (e.g. tabulating the study intervention characteristics and comparing against the planned groups for each synthesis (item #5)). | 5, Table 1 |
|  | 13b | Describe any methods required to prepare the data for presentation or synthesis, such as handling of missing summary statistics, or data conversions. | NA |
|  | 13c | Describe any methods used to tabulate or visually display results of individual studies and syntheses. | 5 |
|  | 13d | Describe any methods used to synthesize results and provide a rationale for the choice(s). If meta-analysis was performed, describe the model(s), method(s) to identify the presence and extent of statistical heterogeneity, and software package(s) used. | 5 |
|  | 13e | Describe any methods used to explore possible causes of heterogeneity among study results (e.g. subgroup analysis, meta-regression). | 5 |
|  | 13f | Describe any sensitivity analyses conducted to assess robustness of the synthesized results. | 5 |
| Reporting bias assessment | 14 | Describe any methods used to assess risk of bias due to missing results in a synthesis (arising from reporting biases). | NA |
| Certainty assessment | 15 | Describe any methods used to assess certainty (or confidence) in the body of evidence for an outcome. | NA |
| **RESULTS** | | |  |
| Study selection | 16a | Describe the results of the search and selection process, from the number of records identified in the search to the number of studies included in the review, ideally using a flow diagram. | 5, Figure-1, 2 |
|  | 16b | Cite studies that might appear to meet the inclusion criteria, but which were excluded, and explain why they were excluded. | 5, Table 1 |
| Study characteristics | 17 | Cite each included study and present its characteristics. | Table-1 |
| Risk of bias in studies | 18 | Present assessments of risk of bias for each included study. | Table S3 |
| Results of individual studies | 19 | For all outcomes, present, for each study: (a) summary statistics for each group (where appropriate) and (b) an effect estimate and its precision (e.g. confidence/credible interval), ideally using structured tables or plots. | Table 1, 3 |
| Results of syntheses | 20a | For each synthesis, briefly summarise the characteristics and risk of bias among contributing studies. | 5 |
|  | 20b | Present results of all statistical syntheses conducted. If meta-analysis was done, present for each the summary estimate and its precision (e.g. confidence/credible interval) and measures of statistical heterogeneity. If comparing groups, describe the direction of the effect. | 5, 6 |
|  | 20c | Present results of all investigations of possible causes of heterogeneity among study results. | 5, 6 |
|  | 20d | Present results of all sensitivity analyses conducted to assess the robustness of the synthesized results. | 7 |
| Reporting biases | 21 | Present assessments of risk of bias due to missing results (arising from reporting biases) for each synthesis assessed. | NA |
| Certainty of evidence | 22 | Present assessments of certainty (or confidence) in the body of evidence for each outcome assessed. | NA |
| **DISCUSSION** | | |  |
| Discussion | 23a | Provide a general interpretation of the results in the context of other evidence. | 8 |
|  | 23b | Discuss any limitations of the evidence included in the review. | 8 |
|  | 23c | Discuss any limitations of the review processes used. | 8 |
|  | 23d | Discuss implications of the results for practice, policy, and future research. | 9 |
| **OTHER INFORMATION** | | |  |
| Registration and protocol | 24a | Provide registration information for the review, including register name and registration number, or state that the review was not registered. | 4 |
|  | 24b | Indicate where the review protocol can be accessed, or state that a protocol was not prepared. | 4 |
|  | 24c | Describe and explain any amendments to information provided at registration or in the protocol. | NA |
| Support | 25 | Describe sources of financial or non-financial support for the review, and the role of the funders or sponsors in the review. | 10 |
| Competing interests | 26 | Declare any competing interests of review authors. | 10 |
| Availability of data, code and other materials | 27 | Report which of the following are publicly available and where they can be found: template data collection forms; data extracted from included studies; data used for all analyses; analytic code; any other materials used in the review. | 10 |

**Table S2.** The adjusted search terms as per searched electronic databases

| Database | No | Electronic cigarette and sleep duration, sleep issues and Insomnia | Results |
| --- | --- | --- | --- |
|  | |  | |
| PubMed | #1 | "Sleep*" OR "Sleeping Habit*" OR "Sleep Habit*" OR "Sleepiness Scale" OR "sleep scale" OR "Sleep disorder*" OR "abnormal dream*" OR "sleep disturbance*" OR "Sleep–wake disorder*" OR "Sleep apnea syndrome" OR "Parasomnia" OR "Sleep initiation and maintenance Disorder*" OR "Dyssomnia*" OR "Insomnia*" OR "sleep*" | 322996 |
|  | #2 | "vaping" OR "vaper*" OR "e-cigarette*" OR "electronic cigarette*" OR "e-cigs" OR "vape users" OR "e-cigarette users" OR "vape" OR "vape*" OR "e-cig*" OR "electronic nicotine" | 14471 |
|  | #3 | #1 AND #2 | 291 |
| EMBASE | #1 | sleep:ab,ti,kw OR 'sleeping habit*':ab,ti,kw OR 'sleep habit*':ab,ti,kw OR 'sleepiness scale':ab,ti,kw OR 'sleep scale':ab,ti,kw OR 'sleep disorder*':ab,ti,kw OR 'abnormal dream*':ab,ti,kw OR 'sleep disturbance*':ab,ti,kw OR 'sleep–wake disorder*':ab,ti,kw OR 'sleep apnea syndrome':ab,ti,kw OR parasomnia:ab,ti,kw OR 'sleep initiation and maintenance disorder*':ab,ti,kw OR dyssomnia:ab,ti,kw OR insomnia:ab,ti,kw OR 'disorders of initiating and maintaining sleep':ab,ti,kw | 380995 |
|  | #2 | 'vaping':ab,ti OR 'vaper*':ab,ti OR 'e-cigarette*':ab,ti OR 'e-cig*':ab,ti OR 'vape user*':ab,ti OR 'e-cigarette user*' OR 'electronic cigarette*':ab,ti OR 'vape':ab,ti | 15079 |
|  |  |  |  |
|  | #3 | #1 AND #2 | 171 |
| WOS  advanced | #1 | (TI=(Sleep* OR “Sleeping Habit*” OR “Sleep Habit* ” OR “Sleepiness Scale ”OR “sleep scale ” OR “Sleep disorder*” OR “abnormal dream*” OR “sleep disturbance *” OR “Sleep–wake disorder*” OR “Sleep apnea syndrome” OR Parasomnia OR “Sleep initiation and maintenance Disorder*” OR Dyssomnia* OR Insomnia* OR “Disorders of initiating and maintaining sleep”)) OR AB=(Sleep* OR “Sleeping Habit*” OR “Sleep Habit* ” OR “Sleepiness Scale ”OR “sleep scale ” OR “Sleep disorder*” OR “abnormal dream*” OR “sleep disturbance *” OR “Sleep–wake disorder*” OR “Sleep apnea syndrome” OR Parasomnia OR “Sleep initiation and maintenance Disorder*” OR Dyssomnia* OR Insomnia* OR “Disorders of initiating and maintaining sleep”) | 279777 |
|  | #2 | (TI=("vaping" OR "vaper*" OR "e-cigarette*" OR "electronic cigarette*" OR "e-cig*" OR "vape user*" OR "e-cigarette user*")) OR AB=("vaping" OR "vaper*" OR "e-cigarette*" OR "electronic cigarette*" OR "e-cig*" OR "vape user*" OR "e-cigarette user*") | 11736 |
|  |  |  |  |
|  | #3 | #1 AND #2 |  |

**Table S3.** Quality assessment using Newcastle-Ottawa Scale

| **STUDY** | **SELECTION (max 4 points)** | | | | **COMPARABILITY (max 2 points)** | **OUTCOME (max 3 points)** | | | **SCORE (out of 6)** |
| --- | --- | --- | --- | --- | --- | --- | --- | --- | --- |
|  | Representativeness | Selection | Ascertainment | Demonstration of the outcome of interest was not present at start of study | Comparability the basis of the design or analysis | Assessment of outcome | Was follow-up long enough for outcomesto occur? | Adequacy of the follow-up |  |
| Baiden 2023 (1) | 1 | 1 | 1 | NA | 1 | 1 | NA | NA | 5 |
| Brett 2020 (2) | 0 | 1 | 1 | NA | 1 | 1 | NA | NA | 4 |
| Kianersi 2021 (3) | 1 | 1 | 1 | NA | 2 | 1 | NA | NA | 6 |
| Lee 2021 (4) | 1 | 1 | 1 | NA | 1 | 1 | NA | NA | 5 |
| Mahamid 2022(5) | 1 | 1 | 1 | NA | 1 | 1 | NA | NA | 5 |
| Merianos 2021 (6) | 0 | 1 | 1 | NA | 1 | 1 | NA | NA | 4 |
| Riehm 2019 (7) | 1 | 1 | 1 | NA | 2 | 1 | NA | NA | 6 |
| So 2021 (8) | 1 | 1 | 1 | NA | 1 | 1 | NA | NA | 5 |
| Thepthien 2023 (9) | 1 | 1 | 1 | NA | 1 | 1 | NA | NA | 5 |
| Wang 2024 (10) | 1 | 1 | 1 | NA | 2 | 1 | NA | NA | 6 |
| Wiener 2020 (11) | 1 | 1 | 1 | NA | 1 | 1 | NA | NA | 5 |
| Wilson 2024 (12) | 1 | 1 | 1 | NA | 1 | 1 | NA | NA | 5 |
| You 2023 (13) | 1 | 1 | 1 | NA | 1 | 1 | NA | NA | 5 |
| Zhu 2023 (14) | 1 | 1 | 1 | NA | 2 | 1 | NA | NA | 6 |

**Bibliography**

1. Baiden P, Spoor SP, Nicholas JK, Brown FA, LaBrenz CA, Spadola C. Association between use of electronic vaping products and insufficient sleep among adolescents: Findings from the 2017 and 2019 YRBS. Sleep Medicine. 2023;101:19-27.

2. Brett EI, Miller MB, Leavens ELS, Lopez SV, Wagener TL, Leffingwell TR. Electronic cigarette use and sleep health in young adults. Journal of Sleep Research. 2019;29(3).

3. Kianersi S, Zhang Y, Rosenberg M, Macy JT. Association between e-cigarette use and sleep deprivation in U.S. Young adults: Results from the 2017 and 2018 Behavioral Risk Factor Surveillance System. Addictive Behaviors. 2021;112.

4. Lee BG, Lee H. Associations between Cigarette and Electronic Cigarette Use and Sleep Health in Korean Adolescents: An Analysis of the 14th (2018) Korea Youth Risk Behavior Surveys. Journal of Korean Academy of Nursing. 2021;51(3).

5. Mahamid F, Bdier D, Damiri B. Energy drinks, depression, insomnia and stress among Palestinians: The mediating role of cigarettes smoking, electronic cigarettes and waterpipe. Journal of Ethnicity in Substance Abuse. 2022:1-16.

6. Merianos AL, Jandarov RA, Choi K, Fiser KA, Mahabee-Gittens EM. Combustible and electronic cigarette use and insufficient sleep among U.S. high school students. Preventive Medicine. 2021;147.

7. Riehm KE, Rojo‐Wissar DM, Feder KA, Mojtabai R, Spira AP, Thrul J, et al. E‐cigarette use and sleep‐related complaints among youth. Journal of Adolescence. 2019;76(1):48-54.

8. So CJ, Meers JM, Alfano CA, Garey L, Zvolensky MJ. Main and Interactive Effects of Nicotine Product Type on Sleep Health Among Dual Combustible and E‐Cigarette Users. The American Journal on Addictions. 2020;30(2):147-55.

9. Thepthien B-o, Tinn CS, Sharma R. Establishing the Association Between Traditional Tobacco, E-cigarette and Dual Use and Mental Health Problems Among High School Students: Results from a 2022 Behavioral Surveillance Survey. International Journal of Mental Health and Addiction. 2023.

10. Wang S, Nandy RR, Rossheim ME. Associations between e-cigarette use and sleep health among adults in the United States, NHANES 2015–2018. Sleep Medicine. 2024;114:220-8.

11. Wiener RC, Waters C, Bhandari R, Trickett Shockey AK, Alshaarawy O. The Association of Sleep Duration and the Use of Electronic Cigarettes, NHANES, 2015-2016. Sleep Disorders. 2020;2020:1-12.

12. Wilson OWA, Bullen C, Duffey M, Bopp M. The association between vaping and health behaviors among undergraduate college students in the United States. Journal of American College Health. 2022;72(5):1360-4.

13. You MA, Choi J, Son YJ. Associations of dual use of tobacco cigarettes and e‐cigarettes, sleep duration, physical activity and depressive symptoms among middle‐aged and older Korean adults. Nursing Open. 2023;10(6):4071-82.

14. Zhu H, Wu M. A cross-sectional study on the relationship between electronic cigarette and combustible cigarette use with obstructive sleep apnea among U.S. adults: result from NHANES 2015–2018. Archives of Public Health. 2023;81(1).
